# A Simple Scoring Method for Predicting the Low Risk of Persistent Acute Kidney Injury in Critically Ill Adult Patients

**DOI:** 10.1038/s41598-020-62479-w

**Published:** 2020-03-31

**Authors:** Ryo Matsuura, Masao Iwagami, Hidekazu Moriya, Takayasu Ohtake, Yoshifumi Hamasaki, Masaomi Nangaku, Kent Doi, Shuzo Kobayashi, Eisei Noiri

**Affiliations:** 1 Department of Nephrology and Endocrinology, The University of Tokyo Hospital, Tokyo, Japan; 2 Department of Hemodialysis and Apheresis, The University of Tokyo Hospital, Tokyo, Japan; 3 Department of Health Services Research, Faculty of Medicine, University of Tsukuba, Ibaraki, Japan; 4 Department of Nephrology, Immunology, and Vascular Medicine, Kidney Disease and Transplant Center, Shonan Kamakura General Hospital, Kamakura, Japan; 5 Department of Emergency and Critical Care Medicine, The University of Tokyo Hospital, Tokyo, Japan; 6 National Center Biobank Network, National Center for Global Health and Medicine, Tokyo, Japan

**Keywords:** Acute kidney injury, Predictive markers

## Abstract

The renal angina index has been proposed to identify patients at high risk of persistent AKI, based on slight changes in serum creatinine and patient conditions. However, a concise scoring method has only been proposed for pediatric patients, and not for adult patients yet. Here, we developed and validated a concise scoring method using data on patients admitted to ICUs in 21 Japanese hospitals from 2012 to 2014. We randomly assigned to either discovery or validation cohorts, identified the factors significantly associated with persistent AKI using a multivariable logistic regression model in the discovery cohort to establish a scoring system, and assessed the validity of the scoring in the validation cohort using receiver operating characteristic analysis and the calibration slope. Among 8,320 patients admitted to the ICUs, persistent AKI was present in 1,064 (12.8%) patients. In the discovery cohort (n = 4,151), ‘hyperbilirubinemia’, ‘sepsis’ and ‘ventilator and/or vasoactive’ with small changes in serum creatinine were selected to establish the scoring. In the validation cohort (n = 4,169), the predicting model based on this scoring had a c-statistic of 0.79 (95%CI, 0.77–0.81) and was well calibrated. In conclusion, we established a concise scoring method to identify potential patients with persistent AKI, which performed well in the validation cohort.

## Introduction

Acute kidney injury (AKI) represents a significant clinical burden with a high mortality rate of 20% for critically ill patients worldwide^1,2^. Persistent AKI patients for more than two days are more likely to have higher mortality^3,4^. Unfortunately, there is no effective treatment for developed AKI and physicians should take actions before developing AKI, such as avoiding nephrotoxic agents, managing volume status and stabilizing hemodynamic status to prevent AKI development. Therefore, early detection and intervention for patients at high risk of persistent AKI is crucial for preventing adverse consequences. However, clinical risk scores for persistent AKI have not yet been developed, partly because the risk factors contributing to persistent AKI are not well known^5^.

Checking serum creatinine levels alone is suboptimal for the early detection of persistent AKI due to the delayed elevation of serum creatinine after a renal insult and the decrease in creatinine production in critically ill adult patients^6,7^. Thus, other clinical markers are required to identify patients at high risk of persistent AKI.

The renal angina index (RAI), based on small changes in serum creatinine and patient conditions, has recently been proposed to identify critically ill patients at higher risk of persistent AKI^8^. The concept of “renal angina” has come into use to highlight the characteristics of renal injury in analogy to the concept of “angina pectoris”, which is used to increase the suspicion of acute coronary syndrome in cardiology. Therefore, the RAI is expected to detect early signs of persistent AKI^8^. In previous reports, this concept of RAI was operationalized and validated in pediatric patients using a simple scoring system consisting of changes in creatinine clearance and fluid overload, stem cell transplantation, and ventilator and/or inotropy^9–11^.

However, the RAI above cannot be used for adult patients because it is determined based on pediatric population, which is totally different from adult patients in respect of comorbidities and risk factors for AKI. In addition, such a concise scoring system has not yet been well developed and validated in adult patients. The aim of this study is to evaluate the association between various risk factors and persistent AKI in adult ICU patients and to develop and validate the concise scoring system using a Japanese multicenter database.

## Materials and Methods

### Data source and study population

We used data on patients admitted to ICUs in 21 hospitals belonging to the Tokushukai Medical Group that participated in the diagnosis procedure combination system between 2012 and 2014^12^. The details of this medical database have been explained previously^12^. The current study is a sub-analysis of data used in a previous study on AKI incidence and outcomes^13^.

The study was conducted in accordance with the Declaration of Helsinki and was approved by the Tokushukai Group Joint Ethics Committee. This committee determined that informed consent was waived because the data were anonymous.

### Study population, design, and outcome

All patients admitted to the ICUs in 21 hospitals during the 2012 to 2014 period were included. Excluded were those who received renal replacement therapy (RRT), those whose ICU stay was less than 24 hours, those whose serum creatinine was over 4 mg/dL at hospital admission, and those for whom serum creatinine was not measured the following day after ICU admission.

**Figure 1:**
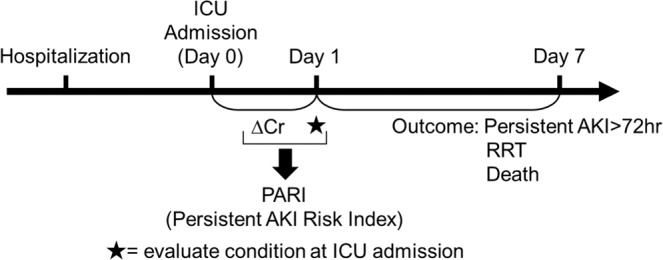
Study Design The PARI was calculated the day after ICU admission based on each patient’s condition at admission to the ICU and the slight changes in serum creatinine (ΔCr) levels between ICU admission and the following day. The primary outcome measure was KDIGO AKI stage 2/3 persisting over three days, and the secondary outcome measures were RRT and death within one week.

After applying the exclusion criteria, the eligible patients were randomly divided into two groups: the discovery cohort and the validation cohort. In the discovery cohort, we identified the factors that were significantly associated with the incidence of persistent AKI (the primary outcome) and developed a concise scoring system called as Persistent AKI Risk Index (PARI). Next, the PARI was validated in the validation cohort. All patients were evaluated for the PARI the following day after their ICU admission (Fig. 1).

The primary outcome was persistent AKI, defined as AKI stage 2/3, persisting over three days within one week of ICU admission. AKI is defined based on serum creatinine according to the Kidney Disease: Improving Global Outcomes (KDIGO) guideline. If patients had recurrent AKI, we evaluated whether every episode of AKI was persistent or not. In patients who did not have daily serum creatinine, if they had stage 2/3 AKI on two days in sequence and then no serum creatinine on the third day, persistent AKI was determined by whether or not they had stage 2/3 AKI on the fourth day. The secondary outcomes were the initiation of RRT and death within one week of ICU admission.

### Persistent AKI risk index (PARI)

In this study, we use the term “persistent AKI risk index (PARI)” instead of RAI. PARI is a composite of risk factors and clinical signs of AKI. The rationale for the PARI is that patients who have risk factors at differing risk levels for AKI can be identified as high risk for persistent AKI by meeting the differing criteria on clinical signs of AKI, such as small changes in serum creatinine^8^. If a patient has higher risk factors, they require less clinical signs of AKI (very small changes in serum creatinine) early on in order to fulfill renal angina. Similarly, if a patient has fewer risk factors but shows more overt signs of clinical AKI (moderate changes in serum creatinine), renal angina would also be fulfilled. Per the epidemiology of AKI, the risk of AKI increases in a multiplicative fashion with increased risk factors. The incidence of AKI demonstrates fold-increases for higher-risk patients. Thus, just like the previous study^10^, the PARI was calculated by a multiplicative index based on a change in serum creatinine (namely, the creatinine component) and patient conditions (namely, condition component) in reference to the RAI for pediatric ICU patients. The creatinine component was determined based on a univariable logistic regression analysis in the association of the difference in serum creatinine values (ΔCr) between ICU days 0 and 1, and persistent AKI. We selected ΔCr between ICU days 0 and 1 because this timeframe was felt to be beyond the generally accepted window in the clinical practice, allowing time for fluid resuscitation, optimizing hemodynamics, and appropriate treatment such as antibiotics against infection. The condition component was determined based on a multivariable logistic regression analysis including the following risk factors: older age, diabetes mellitus, cardiovascular disease, chronic kidney disease (CKD), hypertension, morbid obesity, hyperbilirubinemia, cerebrovascular accident, cancer, high-risk surgery, nephrotoxic drugs, sepsis, and ventilator or vasoactive. We selected these candidate variables according to the previous study^14,15^. Age, CKD, morbid obesity and hyperbilirubinemia were diagnosed based on patients’ medical charts including vital signs, and all measured inpatient and outpatient laboratory values. Diabetes mellitus, cardiovascular disease, hypertension, cerebrovascular accident, and cancer were diagnosed based on the records of the attending physician at the time of discharge using International Classification Disease, 10th revision (ICD-10) codes and medication information recorded at hospital admission. High-risk surgery, nephrotoxic drugs, sepsis and use of ventilator and/or vasoactive drugs were diagnosed based on the records by the attending physician at the time of discharge using ICD-10 codes, procedures including ventilator and type of surgery, and the use of intravenous drugs. The definition of these factors in the database is described in detail in Supplemental Table 1. Among these factors, those significantly associated with persistent AKI in the multivariable logistic regression analysis were assigned risk scores according to the odds ratio of the multivariable logistic regression. The condition score was calculated as the sum of the risk scores. If a patient did not have any of these factors, the condition score was 1.

### Statistical analyses

Continuous variables were described as means and standard deviations; categorical variables were expressed with frequencies and proportions. In determining the creatinine component of the PARI in the discovery cohort, unadjusted odds ratios and 95% confidence intervals (CIs) were calculated using a univariable logistic regression analysis. In determining the condition component of the PARI, adjusted odds ratios and 95% CIs were calculated using a multivariable logistic regression analysis including the aforementioned risk factors for persistent AKI. We repeated the analysis in the same way for patients with the first seven days’ serum creatinine available.

The validity of the PARI, ΔCr, and the sequential organ failure (SOFA) score were all evaluated using a receiver operating characteristic curve analysis in the discovery and validation cohorts. Confidence intervals of the area under the curve (AUC) were generated with a bootstrap method (2,000 replications). The comparison of AUC was done using the DeLong method to prove the superiority of the PARI to ΔCr only, and the illness severity score for predicting outcome. The optimal cut-off of the PARI was determined using Youden’s index^16^. The calibration of the model based on the PARI was also assessed by the calibration slope^17,18^. All tests in this report were performed at a nominal significance level of α = 0.05. Calculations were conducted using statistical analysis software (R version 3.4.3, R Development Core Team, Vienna, Austria).

## Results

### Baseline characteristics and outcomes

**Figure 2:**
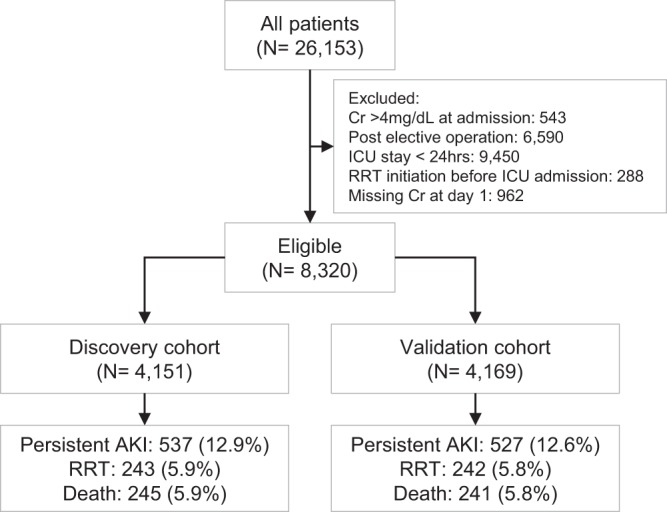
The Patient Flow.

We identified 8,320 eligible patients who were admitted to the ICUs of 21 hospitals between 2012 and 2014, including 4,151 patients in the discovery cohort and 4,169 patients in the validation cohort (Fig. 2). The number and proportion of patients with pre-admission serum creatinine available totaled 7,222 (86.8%) overall, including 3,628 (87.4%) in the discovery cohort and 3,594 (86.2%) in the validation cohort. The SOFA scores in the discovery and validation cohorts were 5.5 ± 3.0 and 5.4 ± 3.0. Within one week of ICU admission, the persistent AKI (primary outcome), RRT, and death (secondary outcomes) occurred in 537 (12.9%), 243 (5.9%), and 245 (5.9%) patients in the discovery cohort, and in 527 (12.6%), 242 (5.8%), and 241 (5.8%) patients in the validation cohort. There were no significant differences in the patients’ backgrounds and the outcome between the discovery and validation cohorts (Table 1).

**Table 1:** Baseline characteristics in the discovery and validation cohort.

	Discovery cohort (N = 4,151)	Validation cohort (N = 4,169)
Age (yr.)	70.8 (14.3)	70.7 (14.6)
Male	2,476 (59.6%)	2,462 (59.1%)
Diabetes mellitus	1,036 (25.0%)	992 (23.8%)
Hypertension	2,217 (53.4%)	2,183 (52.4%)
Cardiovascular disease	2,411 (58.1%)	2,289 (54.9%)
Cerebrovascular disease	856 (20.6%)	818 (19.6%)
Baseline Cr (mg/dL)	0.8 (0.3)	0.8 (0.4)
Baseline eGFR (mL/min/1.73m^2^)	89.9 (88.0)	90.4 (70.4)
CKD (<60 mL/min/1.73m^2^)	1,042 (25.1%)	1,023 (24.5%)
Pre-admission baseline Cr available (N/%)	3,628 (87.4%)	3,594 (86.2%)
Hyperbilirubinemia (N/%)	255 (6.1%)	240 (5.8%)
**Causes of admission (N/%)**		
Cardiovascular	2,645 (63.7%)	2,584 (62.0%)
Endocrinology	75 (1.8%)	61 (1.5%)
Gastrointestinal	386 (9.3%)	412 (9.9%)
Malignancy	81 (2%)	94 (2.3%)
Neurological	37 (0.9%)	60 (1.4%)
Respiratory	321 (7.7%)	332 (8%)
Others	606 (14.6%)	626 (15%)
SOFA score	5.5 ± 3.0	5.4 ± 3.0
Postoperative (N/%)	1,951 (47.0%)	2,052 (49.2%)
Vasopressors (N/%)	1,834 (44.2%)	1,846 (44.3%)
Ventilators (N/%)	904 (21.8%)	879 (21.1%)
Sepsis (N/%)	2,128 (51.3%)	2,199 (52.7%)
Nephrotoxin exposure	493 (11.9%)	492 (11.8%)
ICU stay (days)	8.5 (12.8)	8.4 (10.2)
**AKI stage at admission**		
No AKI	3,127 (75.3%)	3,145 (75.4%)
Stage I	622 (15%)	629 (15.1%)
Stage II	262 (6.3%)	267 (6.4%)
Stage III	140 (3.4%)	128 (3.1%)

The data was not statistically different between the two groups. The data is shown as N (%) or mean (standard deviation). AKI, acute kidney injury; Cr, creatinine; eGFR, estimated glomerular filtration rate; CKD, chronic kidney disease; ICU, intensive care unit.

### The development of the PARI in the discovery cohort

In the evaluation of the association of ΔCr between ICU days 0 and 1 with persistent AKI, the unadjusted odds ratio for the incidence of persistent AKI was ΔCr ≥ 0.4 mg/dL, 10.7 (95% CI 8.55–13.5); ΔCr ≥ 0.3 mg/dL, 3.93 (95% CI 2.68–5.67); and ΔCr ≥ 0.2 mg/dL, 2.15 (1.47–3.07) (Supplemental Table 2).

As for the condition component, a multivariate logistic regression analysis revealed that hyperbilirubinemia (adjusted odds ratio [aOR] 1.45, 95% CI 1.03–2.03), sepsis (aOR 1.53, 95% CI 1.03–2.03), and ventilator and/or vasoactive (aOR 3.51, 95% CI 2.85–4.35) were significantly associated with persistent AKI whereas other factors were not significant (Supplemental Table 3).

Therefore, the creatinine score was determined as follows: those with ΔCr ≥ 0.4 mg/dL, 10 points; those with ΔCr ≥ 0.3 mg/dL, 4 points; those with ΔCr ≥ 0.2 mg/dL, 2 points; and those with ΔCr < 0.2 mg/dL, 1 point. As for the condition component, hyperbilirubinemia, sepsis, and ventilator and/or vasoactive were assigned with scores of 2, 2, and 4, respectively.

**Figure 3:**
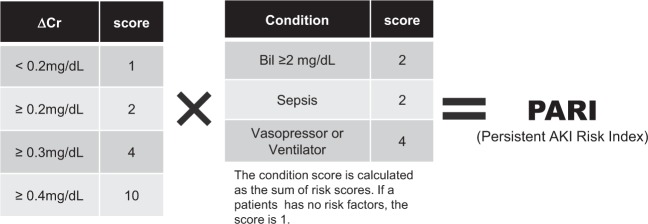
The Components of the PARI. The creatinine score and each patient’s condition were determined as depicted. The creatinine score was calculated by ΔCr between ICU admission and the next day as follows: those with ΔCr ≥ 0.4 mg/dL, 10 points; those with ΔCr ≥ 0.3 mg/dL, 4 points; those with ΔCr ≥ 0.2 mg/dL, 2 points; and those with ΔCr < 0.2 mg/dL, 1 point. As for the condition score, ventilator and/or vasoactive, hyperbilirubinemia, and sepsis were assigned scores of 4, 2, and 2, respectively. The condition score is calculated as the sum of the risk score. If a patient did not have these risk factors, the condition score was 1. The PARI score was defined as the condition score multiplied by the creatinine score and consisted of 1, 2, 4, 6, 8, 10, 12, 16, 20, 24, 32, 40, 60, and 80.

Finally, the PARI score was developed as a multiplication of the creatinine component (score 1, 2, 4, or 10) and condition component (score 1, 2, 4, 6, or 8) (Fig. 3), providing scores of 1, 2, 4, 6, 8, 10, 12, 16, 20, 24, 32, 40, 60, and 80.

### The performance of the PARI, ΔCr and SOFA score to predict persistent AKI in the discovery cohort

**Figure 4:**
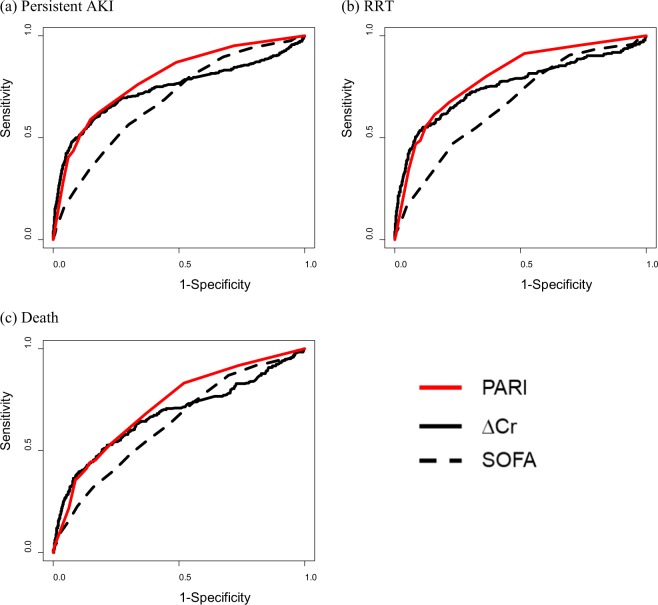
The receiver operating characteristic curves of the PARI, the integrated score, ΔCr and SOFA score for predicting (**a**) persistent AKI, (**b**) RRT and (**c**) Death in the discovery cohort.

In the discovery cohort, the AUC of the PARI for predicting persistent AKI was 0.79 (95%CI, 0.77–0.81), whereas that of RRT and death were 0.80 (95%CI, 0.77–0.83) and 0.72 (95%CI, 0.69–0.75), respectively (Fig. 4, Table 2). The validity measures at different cut-off points are shown in Supplemental Table 4.

**Table 2:** The AUCs of each variable for predicting outcome in the discovery cohort.

	AUC (bootstrap 95% CI)	*P* value vs the PARI
**(a) Persistent AKI**		
The PARI	0.79 (0.77–0.81)	—
ΔCr	0.74 (0.71–0.77)	<0.001
SOFA	0.69 (0.66–0.71)	<0.001
**(b) RRT**		
The PARI	0.80 (0.77–0.83)	—
ΔCr	0.75 (0.72–0.79)	<0.001
SOFA	0.67 (0.64–0.70)	<0.001
**(c) Death**		
The PARI	0.72 (0.69–0.75)	—
ΔCr	0.68 (0.63–0.72)	0.003
SOFA	0.63 (0.60–0.67)	<0.001

These tables described the AUCs for predicting (a) persistent AKI, (b) RRT and (c) death. AUC, the area under the curve; PARI, persistent AKI risk index; SOFA, sequential organ failure score.

On the other hand, the AUCs of ΔCr and the SOFA score for predicting persistent AKI were 0.74 (95%CI, 0.71–0.77) and 0.69 (95%CI, 0.66–0.71) (Fig. 4, Table 2), which were significantly lower than that of the PARI (p < 0.001). The AUCs of ΔCr and the SOFA score for predicting RRT and death were also significantly lower than that of the PARI (Fig. 4, Table 2). This finding suggests that the PARI is superior to both ΔCr and the SOFA score for predicting persistent AKI. The PARI showed a good performance for identifying the potential risk of persistent AKI in the discovery cohort.

### Validation of the PARI

**Figure 5:**
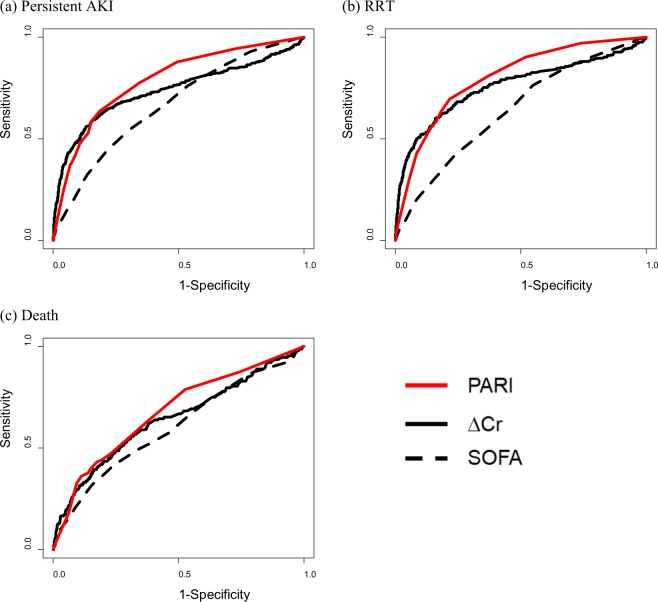
The receiver operating characteristic curves of the PARI, the integrated score, ΔCr and SOFA score for predicting (**a**) persistent AKI, (**b**) RRT and (**c**) Death in the validation cohort.

In the validation cohort, the AUC of the PARI for predicting persistent AKI was 0.79 (95%CI, 0.77–0.81), whereas that of RRT and death were 0.80 (95%CI, 0.77–0.83) and 0.68 (95%CI, 0.64–0.71), respectively (Fig. 5, Table 3). The optimal cut-off of the PARI for predicting the persistent AKI was found to be 8, according to the Youden’s index. The sensitivity, specificity, positive predictive value, and negative predictive value of the PARI for the persistent AKI at this cut-off point (i.e., score ≥8) were 63.8%, 81.3%, 33.0%, and 93.9% respectively (Table 4). The validity measures at different cut-off points are shown in Supplemental Table 5. The risk model, based on the PARI, was well calibrated (calibration slope, 1.06; *P* value = 0.46, Brier score, 0.084, Supplemental Fig. 1).

**Table 3:** The AUCs of each variable for predicting outcome in the validation cohort.

	AUC (bootstrap 95% CI)	*P* value vs the PARI
**(a) Persistent AKI**		
The PARI	0.79 (0.77–0.81)	—
ΔCr	0.74 (0.71–0.76)	<0.001
SOFA	0.67 (0.64–0.69)	<0.001
**(b) RRT**		
The PARI	0.80 (0.77–0.83)	—
ΔCr	0.76 (0.72–0.80)	0.001
SOFA	0.64 (0.61–0.68)	<0.001
**(c) Death**		
The PARI	0.68 (0.64–0.71)	—
ΔCr	0.64 (0.60–0.68)	0.02
SOFA	0.60 (0.56–0.64)	0.001

These tables described the AUCs for predicting (a) persistent AKI, (b) RRT and (c) death. AUC, the area under the curve; PARI, persistent AKI risk index; SOFA, sequential organ failure score.

**Table 4:** The Performance of the PARI for Predicting the Outcome in the validation cohort.

	PARI(+) (n = 1,018)	PARI(−) (n = 3,151)	Sensitivity (%)	Specificity (%)	PPV (%)	NPV (%)
Persistent AKI	336	191	63.8 (59.6–67.7)	81.3 (80.0–82.6)	33.0 (30.9–35.2)	93.9 (93.3–94.6)
RRT	168	74	69.4 (63.2–75.2)	78.4 (77.1–79.7)	16.5 (15.1–18.0)	97.7 (97.2–98.1)
Death	114	127	47.3 (41.1–53.5)	77.0 (75.7–78.3)	11.2 (9.8–12.6)	96.0 (95.5–96.4)

The PARI (+) is defined as ≥8 as determined by a logistic regression model. Numbers in parentheses denote 95% CIs. AKI, acute kidney injury; NPV, negative predictive values; PPV, positive predictive values; PARI, persistent AKI risk index; RRT, renal replacement therapy.

On the other hand, the AUC of ΔCr and SOFA score for predicting persistent AKI were 0.74 (95%CI, 0.71–0.76) and 0.67 (95%CI, 0.64–0.69) (Fig. 5, Table 3), which were significantly lower than the AUC of the PARI (p < 0.001). The AUCs of ΔCr and the SOFA score for predicting RRT and death were also significantly lower than that of the PARI (Fig. 5, Table 3). This finding suggests that the PARI is superior to ΔCr and SOFA score in predicting persistent AKI. The PARI could also perform well in identifying the potential risk of persistent AKI in the validation cohort.

## Discussion

Persistent AKI is a clinical syndrome in ICUs and is associated with poor prognosis^4,19^. An effective therapy for AKI has not yet been established, and therefore, early recognition and care for patients at high risk of persistent AKI is important^20^. The concept of RAI has been proposed in order to detect patients at high risk of persistent AKI^8^. In critically ill children, the RAI has been validated as a screening tool and its predictive ability for persistent AKI has been identified^9,10,21^. We have developed a PARI for adult ICU patients and have found it to be an effective tool to exclude those at low risk of persistent AKI and to identify those at potentially high risk of persistent AKI.

Among the strengths of the PARI is that it is available for excluding patients at low risk of persistent AKI with an easy calculation that can be done at the patient’s bedside. Indeed, we selected only three risk factors (ventilator and/or vasopressor, hyperbilirubinemia (≥2 mg/dL), and sepsis) among various risk factors and developed the PARI so that we could calculate it easily. In addition, the PARI developed in this study is a good predictor for persistent AKI, with an AUC of 0.79.

The PARI does not have an extremely high PPV and cannot be expected to detect those patients at higher risk of persistent AKI. However, renal angina aims to screen patients at risk of subsequent persistent AKI, and it is enough that the scoring has a high NPV, similar to the previous studies^9,10^. The PARI, in our analysis, also has a high NPV and is expected to exclude the low risk of persistent AKI (about 87.5% occupied in ICU) and to identify those at potentially high risk of persistent AKI (about 12.5% occupied in ICU).

We believe that the PARI would be helpful for early identification of those at potential risk of persistent AKI (or to exclude those at low risk of persistent AKI), thereby avoiding further kidney damage. Persistent AKI is associated with a poor prognosis. However, as mentioned earlier, there are no effective treatments for preventing AKI. Early identification of persistent AKI is important to initiate an extended evaluation and management protocol in order to avoid further kidney damage. The Acute Disease Quality Initiative (ADQI) workgroup also recommends that the presence of persistent AKI should prompt a re-assessment of the possible causes of AKI and the reconsideration of treatment options. However, a clinical score for detecting the potential risk of persistent AKI has not been validated so far. The PARI can be expected to detect the potential population of patients at high risk of persistent AKI. We would like to propose that PARI is used with AKI biomarkers to stratify the risk of AKI for all adult ICU patients. The risk assessment by combining PARI and biomarkers can provide real-time clinical decision support with respect to fluid management, nephrotoxic medication avoidance, and more confidence in escalating care to include renal replacement therapy, as written in the previous paper^22^. For example, if patients have low PARI score, you provide standard ICU care to them. If a patient has high PARI score, you measure urinary biomarker and assess the risk of persistent AKI. According to the value of urinary biomarker  and urine output, physicians can decide to closely monitor fluid balance, stabilize hemodynamics and provide renal replacement therapy.

Several limitations of this study must be acknowledged. First, this is a retrospective study and it may, therefore, suffer from the inherent bias common to all retrospective studies. Although we conducted this study in multiple centers (the ICUs of 21 private hospitals in Japan), the study participants may not be representative of the general ICU population. The PARI established in our cohort may need to be validated in other Japanese hospitals and ICUs in other countries, although we believe that the concept of establishing the PARI can be applied in various other ICU settings.

Second, the effect of misclassification in the presence of the risk factors for AKI cannot be denied because it was defined based on recorded diagnosis and prescribed drugs in this study. Thus, this misclassification might lead to the finding that the risk factors, such as diabetes, hypertension, and nephrotoxic drugs were not significantly associated with persistent AKI or selected as the components of the PARI.

Third, although we have adjusted for multiple risk factors in the analysis according to the previous study, this study may not control for unmeasured confounding factors such as urine output. A considerable number of patients are diagnosed with AKI solely on urine output-based criteria. It is also recommended that PARI is calculated based on urine output as well as serum creatinine. Therefore, the inclusion of urine output may make it sensitive to detect the risk for AKI and improve the PPV of the PARI to stratify the risk for AKI. The unavailability of urine output is a limitation of this study.

Fourth, those with serum creatinine higher than 4.0 mg/dL were excluded in this study and this exclusion may worse the performance of PARI. However, according to KDIGO guideline, serum creatinine higher than 4.0 mg/dL is automatically classified as AKI stage 3 even if patients are already in CKD with serum creatinine of higher than 4.0 mg/dL. Thus, inclusion of these patients would not absolutely have improved the sensitivity or the positive predictive value of PARI.

In conclusion, this study suggests that the PARI is an inexpensive, simple, and reliable method for identifying potential patients at high risk of persistent AKI. The concept of establishing a PARI may be applicable to various clinical conditions in adult ICU settings for its simplicity and its rapid impact.

## Supplementary information

Supplementary Table 1-5 & Figure 1.

## Data Availability

All data generated or analyzed during this study are included in this published article.
