# Supplementary material for: A Simple Scoring Method for Predicting the Low Risk of Persistent Acute Kidney Injury in Critically Ill Adult Patients
Source: Sci Rep. 2020 Mar 31;10:5726. doi: 10.1038/s41598-020-62479-w (PMC7109040; doi:10.1038/s41598-020-62479-w)
Supplement: Supplementary file 1 — Supplementary Table 1-5 & Figure 1. [file 41598_2020_62479_MOESM1_ESM.pdf]

A Simple Scoring Method for Predicting the Low Risk of Persistent Acute Kidney Injury in Critically Ill Adult Patients

Ryo Matsuura, M.D., Masao Iwagami, M.D., M.P.H., MSc., Ph.D., Hidekazu Moriya, M.D., Takayasu Ohtake, M.D., Ph.D., Yoshifumi Hamasaki, M.D., Ph.D., Masaomi Nangaku, M.D., Ph.D., Kent Doi, M.D., Ph.D., Shuzo Kobayashi, M.D., Ph.D., Eisei Noiri, M.D., Ph.D.

Table of Contents

Supplemental Table 1. The risk factors for AKI.

Supplemental Table 2: The unadjusted odds ratios of ΔCr between ICU admission and the following day

Supplemental Table 3: The adjusted odds ratio of each factor for outcome in the discovery cohort.

Supplemental Table 4: The predictive performance of each PARI cut-off in the discovery cohort.

Supplemental Table 5: The predictive performance of each PARI cut-off in the validation cohort.

Supplemental Figure 1: The calibration plot of the PARI in the validation cohort.

Supplemental Table 1: **The risk factors for AKI (adapted and modified from reference** (1)**)**

Older age	≥70 yrs
Diabetes mellitus	History of diabetes mellitus
Cardiovascular disease	History of angina pectoris, coronary artery disease, myocardial infarction, heart failure, or peripheral vascular disease
Chronic kidney disease	Baseline eGFR <60mL/min/1.73m^2
Hypertension	History of hypertension or patients receiving chronic antihypertensive medication
Morbid obesity	BMI >30.0 kg/m^2
Hyperbilirubinemia	Serum total bilirubin >2.0 mg/dL
Cerebrovascular accident	History of any type of cerebrovascular accident
Cancer	Active cancer (patients not in remission and without surgical care)
High - risk surgery	Cardiac surgery (valvular or coronary artery bypass grafting), aortic surgery, hepatobiliary surgery (excluding cholecystectomy)
Nephrotoxin exposure	Amphotericin B, aminoglycosides, vancomycin, and non - steroidal anti - inflammatory drugs (NSAIDs) excluding aspirin given prior to or at ICU admission.
Sepsis	Existence of infection treated with antibiotics and life-threatening organ dysfunction identified as an acute change in total SOFA score ≥2.
Ventilator and/or vasopressor	The use of ventilator and/or vasoactive drugs such as norepinephrine, epinephrine, dobutamine and dopamine.

Supplemental Table 2. The unadjusted odds ratios of ΔCr between ICU admission and the following day

ΔCr (mg/dL)	Odds Ratio
<0.1	Reference
≥0.1	0.93 (0.61–1.35)
≥0.2	2.15 (1.47–3.07)
≥0.3	3.93 (2.68–5.67)
≥0.4	10.7 (8.55–13.5)

Supplemental Table 3. The adjusted odds ratio of each factor for outcome in the discovery cohort. The ventilator and/or vasoactive factor was shown to be the highest risk factor. The hyperbilirubinemia and sepsis factors represent moderate risk of persistent AKI.

Condition	Odds ratio
Age ≥70	1.16 (0.94–1.42)
Diabetes mellitus	1.07 (0.86–1.32)
Cardiovascular disease	0.84 (0.67–1.05)
CKD	1.20 (0.97–1.49)
Hypertension	0.80 (0.66–0.98)
Morbid obesity	0.94 (0.56–1.50)
Hyperbilirubinemia (≥2.0 mg/dL)	1.45 (1.03–2.03)
Cerebrovascular accident	0.70 (0.53–0.91)
Cancer	0.85 (0.61–1.17)
High-risk surgery	1.20 (0.96–1.49)
Nephrotoxic drugs	0.81 (0.60–1.09)
Sepsis	1.53 (1.25–1.89)
Ventilator or vasoactive	3.51 (2.85–4.35)

Supplemental Table 4: The predictive performance of each PARI cut-off in the discovery cohort.

Cutoff	Number (N)	Persistent AKI (N)	Sensitivity (%)	Specificity (%)	PPV (%)	NPV (%)	Youden's Index
≥1	4151	537	100.0	0.0	12.9	N/C	0.00
≥2	3107	511	95.2	28.2	16.4	97.5	0.23
≥4	2232	467	87.0	51.2	20.9	96.4	0.38
≥6	1619	409	76.2	66.5	25.3	94.9	0.43
≥8	1009	337	62.8	81.4	33.4	93.6	0.44
≥10	846	316	58.8	85.3	37.4	93.3	0.44
≥12	765	294	54.7	87.0	38.4	92.8	0.42
≥16	671	278	51.8	89.1	41.4	92.6	0.41
≥20	620	263	49.0	90.1	42.4	92.2	0.39
≥24	517	233	43.4	92.1	45.1	91.6	0.36
≥32	440	219	40.8	93.9	49.8	91.4	0.35
≥40	434	217	40.4	94.0	50.0	91.4	0.34
≥60	294	153	28.5	96.1	52.0	90.0	0.25
≥80	20	11	2.0	99.8	55.0	87.3	0.02

Supplemental Table 5. The predictive performance of each PARI cut-off in the validation cohort.

Cutoff	Number (N)	Persistent AKI (N)	Sensitivity (%)	Specificity (%)	PPV (%)	NPV (%)	Youden's Index
≥1	4169	527	100	0	12.6	N/C	0
≥2	3139	497	94.3	27.5	15.8	97.1	0.22
≥4	2259	462	87.7	50.7	20.5	96.6	0.38
≥6	1654	408	77.4	65.8	24.7	95.3	0.43
≥8	1018	336	63.8	81.3	33.0	93.9	0.45
≥10	864	309	58.6	84.8	35.8	93.4	0.43
≥12	786	278	52.8	86.1	35.4	92.6	0.39
≥16	685	260	49.3	88.3	38.0	92.3	0.38
≥20	638	250	47.4	89.3	39.2	92.2	0.37
≥24	532	218	41.4	91.4	41.0	91.5	0.33
≥32	452	198	37.6	93.0	43.8	91.1	0.31
≥40	444	197	37.4	93.2	44.4	91.1	0.31
≥60	284	131	24.9	95.8	46.1	89.8	0.21
≥80	27	16	3.0	99.7	59.3	87.7	0.03

Supplemental Figure 1: The calibration plot of the PARI in the validation cohort.

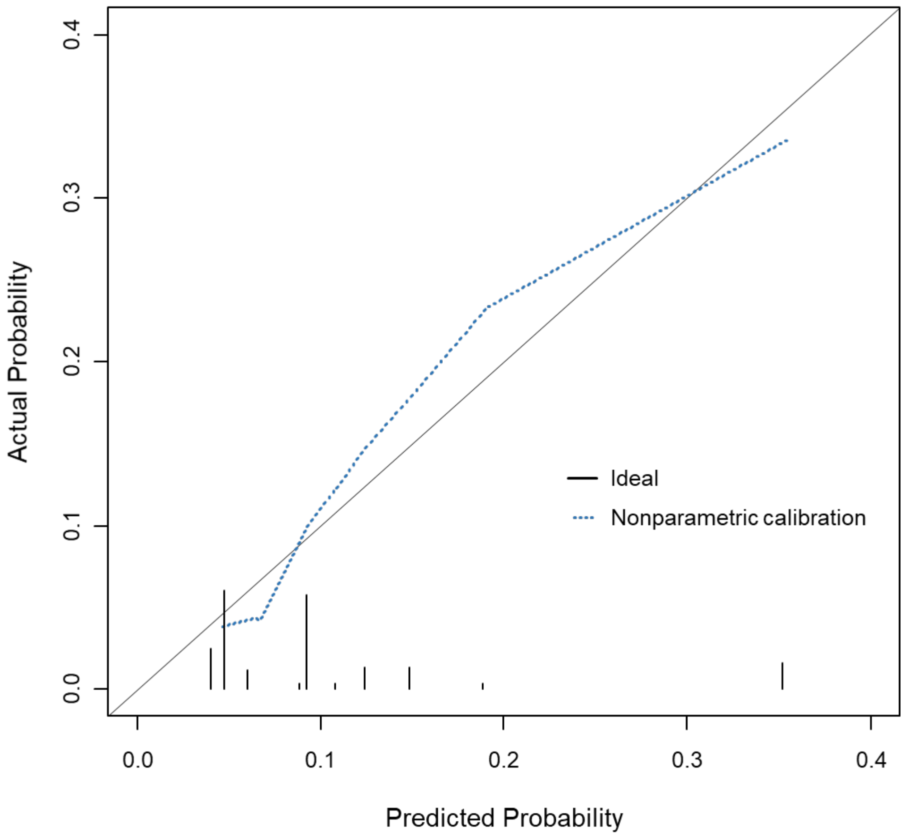

References

1. Chawla LS, Abell L, Mazhari R, Egan M, Kadambi N, Burke HB, Junker C, Seneff MG, Kimmel PL. Identifying critically ill patients at high risk for developing acute renal failure: a pilot study. *Kidney Int* 2005;68:2274–2280.
